# The Application and Molecular Mechanisms of Mitochondria-Targeted Antioxidants in Chemotherapy-Induced Cardiac Injury

**DOI:** 10.3390/cimb47030176

**Published:** 2025-03-07

**Authors:** Chih-Jen Liu, Lu-Kai Wang, Fu-Ming Tsai

**Affiliations:** 1 Division of Cardiology, Department of Internal Medicine, Taipei Tzu Chi Hospital, Buddhist Tzu Chi Medical Foundation, New Taipei City 231, Taiwan; chihjenliu77@gmail.com; 2 Veterinary Diagnostic Division, National Laboratory Animal Center, National Institutes of Applied Research, Taipei City 115, Taiwan; 2407026@narlabs.org.tw; 3 Department of Research, Taipei Tzu Chi Hospital, Buddhist Tzu Chi Medical Foundation, New Taipei City 231, Taiwan

**Keywords:** mitochondria-targeted antioxidants, chemotherapeutic agents, cardiotoxicity, reactive oxygen species

## Abstract

Chemotherapeutic agents play a crucial role in cancer treatment. However, their use is often associated with significant adverse effects, particularly cardiotoxicity. Drugs such as anthracyclines (e.g., doxorubicin) and platinum-based agents (e.g., cisplatin) cause mitochondrial damage, which is one of the main mechanisms underlying cardiotoxicity. These drugs induce oxidative stress, leading to an increase in reactive oxygen species (ROS), which in turn damage the mitochondria in cardiomyocytes, resulting in impaired cardiac function and heart failure. Mitochondria-targeted antioxidants (MTAs) have emerged as a promising cardioprotective strategy, offering a potential solution. These agents efficiently scavenge ROS within the mitochondria, protecting cardiomyocytes from oxidative damage. Recent studies have shown that MTAs, such as elamipretide, SkQ1, CoQ10, and melatonin, significantly mitigate chemotherapy-induced cardiotoxicity. These antioxidants not only reduce oxidative damage but also help maintain mitochondrial structure and function, stabilize mitochondrial membrane potential, and prevent excessive opening of the mitochondrial permeability transition pore, thus preventing apoptosis and cardiac dysfunction. In this review, we integrate recent findings to elucidate the mechanisms of chemotherapy-induced cardiotoxicity and highlight the substantial therapeutic potential of MTAs in reducing chemotherapy-induced heart damage. These agents are expected to offer safer and more effective treatment options for cancer patients in clinical practice.

## 1. Introduction

As the body’s continuously operating pump, the heart requires a constant supply of oxygen and nutrients, and its efficient function depends heavily on mitochondrial activity [1]. Mitochondria, often referred to as the “energy factories” of the cell, generate adenosine triphosphate (ATP) through oxidative phosphorylation, providing the energy needed for cellular processes [2]. Given that the heart must beat continually, cardiomyocytes contain a large number of mitochondria, accounting for about 30% of the cell volume [3]. In addition to providing energy, mitochondria play crucial roles in calcium ion balance, cellular signaling, and the clearance of reactive oxygen species (ROS), all of which are vital for normal heart function [4]. However, mitochondrial dysfunction can lead to insufficient energy production, excessive ROS generation, and cellular damage, which may contribute to cardiac diseases, such as heart failure and arrhythmias [5]. Thus, maintaining mitochondrial health is an important strategy for preventing and treating cardiovascular diseases and may include the use of antioxidants and drugs that promote mitochondrial biogenesis [6].

Mitochondria metabolize glucose and fatty acids to generate ATP through oxidative phosphorylation, and their efficiency is influenced by several factors including oxygen concentration, ADP/ATP ratio, and ROS levels. During increased energy demands such as exercise or stress responses, mitochondria accelerate metabolism [7]. Leakage in the electron transport chain or proton backflow can result in excessive ROS production, mitochondrial damage, and oxidative stress-related diseases [5]. While chemotherapeutic agents and targeted therapies are highly effective against cancer, they can also damage cardiac health, leading to a side effect known as cardiotoxicity [8]. It can manifest in various forms, including myocardial injury, heart failure, and arrhythmias, and can be life threatening [9]. These toxic effects are often related to the mechanisms of drug action, as certain anticancer drugs can damage normal cardiac cells while targeting tumor cells.

Anthracycline chemotherapeutics such as doxorubicin are well known for their high cardiotoxicity [10]. They interfere with DNA replication and transcription to kill cancer cells; however, they also generate ROS within cardiomyocytes, leading to oxidative stress, mitochondrial dysfunction, and cell apoptosis [11]. The cardiotoxicity of these drugs is cumulative, indicating that the higher the total dose, the greater the risk [12]. Targeted therapies such as trastuzumab and tyrosine kinase inhibitors (TKIs) can also affect heart function by disrupting the protective effects of HER2 receptors on cardiomyocytes or damaging endothelial cell function, which can result in hypertension and increased cardiac workload [13].

The development of mitochondria-targeted antioxidants (MTAs) offers a new approach to address chemotherapy-and targeted therapy-induced cardiotoxicity [14]. Excessive ROS can damage mitochondrial DNA, lipids, and proteins, thereby contributing to oxidative stress-related diseases. Consequently, MTA strategies have become a key area of research [14,15]. Traditional antioxidants, such as vitamins C and E, have limited effectiveness owing to their inability to concentrate within the mitochondria [16]. New MTAs are designed to attach antioxidant molecules to positively charged groups (e.g., TPP^+^), which exploit the negatively charged mitochondrial membrane potential to enter the mitochondria and enhance therapeutic efficacy [14]. Moreover, the application of nanotechnology and biocompatible carriers has further improved the mitochondrial targeting of antioxidants, thereby providing potential solutions for treating cardiac toxicity and other diseases [17].

This review focuses on the molecular mechanisms of chemotherapy-induced myocardial injury, exploring the central role of mitochondria in cardiotoxicity and the potential of MTAs in mitigating these side effects. Although chemotherapeutic agents are vital for cancer treatment, their associated cardiotoxicity remains a significant clinical challenge. Thus, MTAs offer a novel and effective strategy for cardioprotection during chemotherapy. By synthesizing recent research, this review highlights the potential of these antioxidants not only in understanding the underlying mechanisms but also in improving the safety of cancer treatments and enhancing patients’ quality of life. The development of cardioprotective drugs holds significant promise for advancing cancer therapy and patient care.

## 2. Types of Chemotherapeutic Agents and Potential Mechanisms of Myocardial Injury

**Figure 1:**
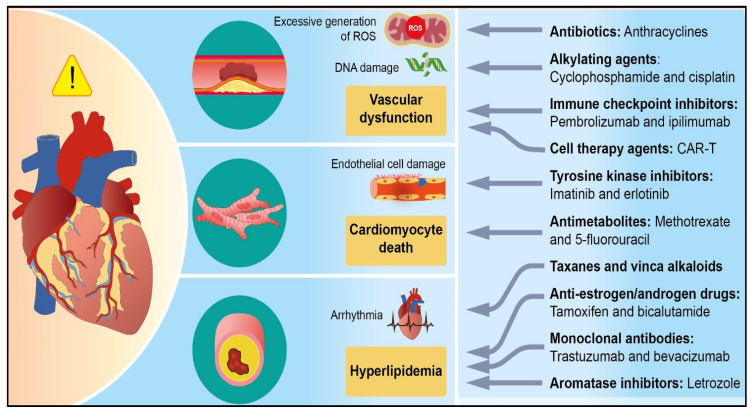
Chemotherapy-induced cardiotoxicity.

Chemotherapeutic agents are classified according to their mechanisms of action and targets. Each category has its own unique way of functioning and corresponding indications. However, various chemotherapeutic drugs that are effective in treating cancer can be toxic to heart cells. The potential primary mechanisms by which these drugs cause cardiac toxicity are summarized in Table 1 and Figure 1 below.

### 2.1. Cytotoxic Drugs

Cytotoxic drugs are a major class of chemotherapeutic agents that primarily target rapidly dividing tumor cells. These drugs interfere with DNA, RNA, and protein syntheses, thereby inhibiting cell growth and proliferation. These include alkylating agents, antimetabolites, and natural product-derived drugs. Although these drugs effectively suppress tumors, they can also affect normal rapidly dividing cells such as those in the bone marrow, gastrointestinal tract, and hair follicles, leading to side effects such as bone marrow suppression, gastrointestinal discomfort, and hair loss.

#### 2.1.1. Alkylating Agents

Alkylating agents are commonly used chemotherapy drugs that exert anticancer effects by forming covalent bonds with DNA, thereby interfering with the DNA function in cancer cells [56]. This disruption inhibited cell division and proliferation, ultimately leading to cell death. The active groups of these drugs can alkylate nitrogenous bases in DNA, particularly at the N7 position of guanine, resulting in single-base alkylation, DNA cross-linking, and other forms of DNA damage. DNA crosslinking disrupts the structure and function of DNA, blocking replication and transcription processes, while single base alkylation may induce mutations or trigger excessive repair mechanisms, leading to cell cycle arrest and apoptosis [57,58]. Since rapidly dividing cancer cells are highly dependent on DNA synthesis, alkylating agents are more toxic to these cells than to quiescent cells, thus providing an anticancer effect. Common alkylating agents include nitrogen mustard, cyclophosphamide, carmustine, and temozolomide, which are widely used to treat lymphomas, leukemia, and certain solid tumors [59,60]. However, these drugs also exhibit toxicity toward normal rapidly dividing cells, such as those in the bone marrow, hair follicles, and gastrointestinal tract, potentially causing bone marrow suppression, hair loss, and gastrointestinal discomfort [61,62]. Furthermore, cancer cells may develop resistance to these drugs by enhancing their DNA repair mechanisms or antioxidant capacity, further challenging the effectiveness of the treatment.

Although alkylating agents are effective in cancer treatment, they may also induce toxicity in cardiac cells through several mechanisms. First, certain metabolites of alkylating agents, such as acrolein (a metabolite of cyclophosphamide), can directly damage cardiomyocytes and endothelial cells [18,19]. Second, alkylating agents can promote ROS generation, leading to oxidative stress, which in turn damages mitochondrial function and induces the apoptosis or necrosis of cardiomyocytes. Moreover, these drugs can bind to DNA, leading to crosslinking and strand breaks, which disrupt DNA replication and transcription, especially in rapidly dividing cardiomyocytes, thus exacerbating the damage [8,20]. They may also induce inflammatory responses by releasing pro-inflammatory mediators that contribute to myocardial inflammation and fibrosis [21]. These combined effects can lead to cardiac toxicity manifesting as heart failure, myocarditis, and pericarditis, which pose a significant threat to heart function and overall health. Therefore, although alkylating agents are the cornerstone of anticancer treatment, their cardiotoxicity must be carefully considered, and strategies to balance therapeutic efficacy and safety should be explored for clinical use.

#### 2.1.2. Antimetabolites

Antimetabolites are essential components of chemotherapy that interfere with cellular metabolism, particularly the synthesis of DNA and RNA, to inhibit the proliferation and survival of cancer cells [63]. These drugs mimic purine or pyrimidine nucleotides and competitively block nucleotide synthesis or integrate into DNA/RNA, causing structural abnormalities [64,65]. Common antimetabolites include 6-mercaptopurine, fluorouracil (5-FU), and methotrexate. For example, 5-FU inhibits thymidylate synthase, thereby blocking DNA synthesis, whereas methotrexate inhibits dihydrofolate reductase, which interferes with purine and pyrimidine biosynthesis [66]. These drugs selectively target rapidly dividing cancer cells but can also harm normal rapidly dividing cells, such as those in the bone marrow and gastrointestinal tract, leading to side effects such as bone marrow suppression, gastrointestinal toxicity, and hair loss [67]. Additionally, cancer cells may develop resistance by altering enzyme structures or increasing the expression of relevant enzymes, necessitating combination therapies to enhance their efficacy [68].

Antimetabolites induce cardiac toxicity. For example, 5-FU has been shown to induce coronary artery spasms, leading to myocardial ischemia, angina, and myocardial infarction [22]. Its metabolites can further inhibit the tricarboxylic acid cycle, damaging the energy metabolism of cardiomyocytes [23,24]. Moreover, antimetabolites can increase ROS production, leading to oxidative stress, mitochondrial dysfunction, and apoptosis or necrosis of cardiomyocytes [25,26]. Oxidative stress can also damage myocardial microvasculature, leading to thrombosis and myocardial ischemia, further impairing heart function [27,28]. These toxic effects can contribute to severe conditions, such as heart failure, and pose a significant threat to patient health. To reduce cardiac toxicity and optimize both treatment safety and efficacy, clinical management should include careful dose control and close monitoring of heart function.

#### 2.1.3. Natural Product-Derived Drugs—Taxanes and Vinca Alkaloids

Taxanes (such as paclitaxel and docetaxel) and vinca alkaloids (such as vincristine and vinblastine) are two classes of anticancer drugs that affect microtubule dynamics [69,70]. Taxanes stabilize microtubule structures, promoting tubulin polymerization and inhibiting depolymerization, thus disrupting mitotic processes in the M phase and eventually inducing apoptosis in cancer cells [71,72]. These drugs are widely used to treat breast, ovarian, and non-small cell lung cancer [73,74,75]. Vinca alkaloids, on the other hand, inhibit the polymerization of tubulin, preventing spindle formation and causing cells to arrest in the metaphase of mitosis, ultimately leading to cell death [76]. They are primarily used to treat lymphomas, leukemia, and some solid tumors [77]. Both drug classes are highly selective for rapidly dividing cancer cells but can cause toxicity in normal cells, especially those that rely on microtubule functions, such as neurons. These side effects include peripheral neuropathy and bone marrow suppression [78]. Additionally, cancer cells can develop resistance through multidrug-resistant proteins or structural changes in tubulin, limiting the efficacy of treatment [70]. To address these issues, researchers have explored drug modifications and combination therapies to enhance the therapeutic outcomes.

Both drug classes can cause cardiac toxicity, although their mechanisms of action are different. The cardiac toxicities of taxanes include arrhythmias, allergic reactions, and microtubule dysfunction [29]. Arrhythmias may present as bradycardia, tachycardia, or atrioventricular block, whereas allergic reactions may cause hypotension and myocardial damage. Microtubule dysfunction further affects normal cardiomyocyte activity [20,30]. The cardiac toxicity of vinca alkaloids is associated with neurotoxicity and bone marrow suppression [31]. Neurotoxicity can impair the autonomic nervous system, leading to arrhythmia and cardiovascular dysfunction. Bone marrow suppression may reduce blood cell counts, indirectly affecting the myocardial oxygen supply and damaging the heart muscle. These effects increase the risk of cardiac toxicity. To minimize side effects, clinical management should include strict dose control and regular monitoring of heart function with early detection and management of related issues. By precisely interfering with microtubule dynamics, both drug classes have become indispensable components of modern cancer chemotherapies.

#### 2.1.4. Natural Product-Derived Drugs—Anthracyclines

Anthracycline antibiotics (e.g., doxorubicin, epirubicin, and daunorubicin) are crucial drugs in cancer chemotherapy that exert anticancer effects through multiple mechanisms that inhibit cancer cell proliferation and promote cell death [79]. Their primary mechanisms include intercalating into the DNA double helix, obstructing DNA replication and transcription, particularly in rapidly dividing cancer cells; stabilizing the complex between topoisomerase II and DNA, resulting in irreversible DNA double-strand breaks and inducing apoptosis; and affecting gene expression and mitochondrial function, triggering intrinsic apoptotic pathways [80]. These multifaceted mechanisms make anthracyclines highly effective for treating breast cancer, lymphomas, leukemia, and various solid tumors, establishing them as the cornerstone of modern cancer treatment [81,82].

However, the cardiac toxicity of anthracyclines limits their clinical use. The primary mechanisms of myocardial injury include the generation of excessive ROS [34], overwhelming the antioxidant capacity of cardiomyocytes and causing cellular damage; chelation of iron [83], which participates in redox reactions, further damaging cell membranes and mitochondrial function; intercalation into DNA, interfering with topoisomerase II activity [84], leading to DNA breaks and initiating apoptosis; and disruption of calcium ion regulation, resulting in calcium imbalance and impaired myocardial contractility [10,32,33,34,85]. These mechanisms can lead to cardiomyopathy, heart failure, and other severe reactions. To mitigate the risk of cardiac toxicity, clinical practice should include strict monitoring of cumulative anthracycline doses and regular assessment of heart function throughout treatment, allowing for the early detection and management of potential cardiac damage. Owing to their multi-target mechanisms, anthracyclines play a critical role in cancer chemotherapy; however, their use must be carefully managed to balance efficacy with safety.

### 2.2. Targeted Therapy Drugs

Targeted therapy drugs are a class of drugs designed to specifically target molecular markers of cancer cells. Unlike traditional chemotherapy drugs, these medications precisely inhibit the growth and spread of cancer cells. Common targeted therapy drugs include tyrosine kinase inhibitors (TKIs) and monoclonal antibodies.

#### 2.2.1. TKIs

TKIs constitute a class of targeted therapies used to treat various cancers. These drugs function by inhibiting the activity of tyrosine kinases, which play a critical role in cellular signaling pathways, especially those involved in cell proliferation, differentiation, and survival [86]. In many tumor cells, the activity of tyrosine kinases is abnormally elevated, contributing significantly to tumor formation and progression. TKIs act through two primary mechanisms: direct inhibition of tyrosine kinase activity and interference with kinase signaling pathways [86,87]. For example, imatinib inhibits the tyrosine kinase activity of the BCR-ABL fusion protein that is abnormally expressed in chronic myelogenous leukemia [88]. Other TKIs, such as erlotinib, gefitinib, and osimertinib, inhibit the EGFR signaling pathway and are commonly used to treat non-small cell lung cancer [89]. TKIs also affect the downstream RAS-RAF-MEK-ERK and PI3K-AKT signaling pathways, enhancing their anticancer effects [90]. Despite the promising results of TKIs in the treatment of various cancers, they may also pose a risk of cardiac toxicity. Certain TKIs, such as afatinib and ponatinib, may damage the heart through mechanisms such as inducing endoplasmic reticulum stress and inflammation, impairing mitochondrial function, affecting endothelial cell function, and prolonging the QT interval (the time from the start of the Q wave to the end of the T wave), which can lead to heart failure or arrhythmias [35,36,37,38]. Osimertinib may cause myocardial injury, potentially due to the instability of its metabolites, oxidative stress, and mitochondrial dysfunction. In plasma, osimertinib degrades easily, potentially generating harmful byproducts that can damage cardiomyocytes and even lead to cardiac dysfunction [91]. To mitigate these side effects, clinical practice requires strict dose control and close monitoring of heart function.

#### 2.2.2. Monoclonal Antibodies

Monoclonal antibody (mAb) anticancer drugs are highly targeted treatments that specifically bind to antigens present on the surface of cancer cells and inhibit tumor cell proliferation, survival, and metastasis. These drugs are designed to precisely target tumor cells, while minimizing their impact on normal cells [92]. The anticancer mechanisms of monoclonal antibodies include inhibiting receptor activation on the surface of cancer cells, triggering immune system attacks on tumor cells, disrupting tumor angiogenesis, and directly inducing cell death [93,94]. For example, trastuzumab binds to the HER2 receptor and inhibits downstream signaling pathways, thereby reducing tumor cell proliferation [95]. It also activates antibody-dependent cellular cytotoxicity (ADCC), prompting natural killer cells in the immune system to attack tumor [96]. Another example is bevacizumab, which inhibits vascular endothelial growth factor (VEGF) to suppress tumor angiogenesis and reduce tumor growth and metastasis, while enhancing the effectiveness of other treatments [97]. Despite their therapeutic benefits, these drugs pose a risk of cardiac toxicity. By inhibiting the HER2 receptor, trastuzumab may interfere with myocardial cell repair, leading to heart function issues [39,40,41]. Recent studies have reported that compared to full-length trastuzumab, Fab fragments exhibit lower toxicity to adult cardiomyocytes while still inhibiting HER2 function. This finding suggests that the FcγRIIA receptor of trastuzumab may play a key role in its cardiotoxicity [98]. Bevacizumab, due to its inhibition of VEGF, increases the risk of hypertension, which may place additional strain on the heart and result in heart failure [42,43].

### 2.3. Immunotherapy Drugs

Immunotherapy drugs primarily activate or enhance a patient’s immune system to fight cancer. These medications include immune checkpoint inhibitors (such as PD-1/PD-L1 and CTLA-4 inhibitors), which relieve the immune suppression exerted by tumor cells, thereby promoting T cell attacks on tumors. Additionally, cytokine therapies (such as IL-2 and interferons) and cancer vaccines are part of immunotherapy, stimulating the immune system to recognize and destroy cancer cells.

#### 2.3.1. Immune Checkpoint Inhibitors (ICIs)

ICIs are a class of immunotherapy drugs that treat cancer by activating the body’s immune system, especially by enhancing the antitumor activity of T cells [99,100]. Tumor cells often exploit immune checkpoint molecules (such as PD-L1) to evade immune surveillance. When PD-L1 binds to the PD-1 receptor on T cells, it inhibits T cell activity, allowing tumor cells to avoid recognition and destruction by the immune system [101]. ICIs, such as PD-1 inhibitors (e.g., nivolumab, pembrolizumab) and PD-L1 inhibitors (e.g., atezolizumab), block these interactions, restore T cell function, and enable them to identify and kill tumor cells [102,103]. Additionally, CTLA-4 inhibitors (e.g., ipilimumab) enhance T cell activity by blocking the binding of CTLA-4 to its ligands [104]. This approach, known as immune checkpoint inhibition therapy, has demonstrated significant effectiveness in treating various cancers, including melanoma, non-small cell lung cancer, and renal cancer [104,105].

Despite its therapeutic benefits, ICI treatment carries a risk of cardiac toxicity, particularly myocarditis. ICIs activate the immune system, leading to an overreaction of T cells, which may attack myocardial cells and cause myocarditis [44,45]. Furthermore, PD-1 and PD-L1 are present in myocardial cells where they maintain immune tolerance. When ICIs interact with these molecules, they disrupt the immune balance in myocardial cells, triggering autoimmune myocardial damage [46,47]. ICIs can also induce systemic inflammatory responses, releasing inflammatory factors like tumor necrosis factor-α (TNF-α) and interleukin-6 (IL-6), which may further damage myocardial cells [48]. Although the incidence of myocarditis caused by ICIs is relatively low, the fatality rate is high (approximately 50%).

#### 2.3.2. Cell Therapy

Cell therapy plays a key role in cancer treatment by enhancing the immune system to destroy tumor cells. Common forms of cell therapy include CAR-T cell, T cell, and natural killer (NK) cell therapies. For example, in CAR T-cell therapy, a patient’s cells are extracted and genetically modified to express chimeric antigen receptors (CARs) on their surface, thereby enabling them to recognize tumor-specific antigens. These modified T cells can precisely attack tumor cells and release cytotoxic molecules to destroy them [106]. CAR-T therapy has shown remarkable effectiveness in treating blood cancers such as acute lymphoblastic leukemia (ALL) and certain types of lymphoma [107]. NK cell therapy, on the other hand, utilizes natural immune cells that can recognize and destroy tumor cells without the need for antigen processing, making it particularly effective against tumor cells with immune evasion mechanisms [108]. Moreover, ICIs can be combined with cell therapy to enhance the immune response of T cells against tumor cells [109]. Another major advantage of cell therapy is its effect on the memory of the immune system. After treatment, enhanced immune cells survive long term in the body and monitor tumor cells, enabling the immune system to quickly recognize and eliminate them in the event of recurrence [110].

Despite its benefits, cell therapy—particularly CAR-T therapy—carries a risk of cardiac toxicity, primarily due to immune system overactivation and excessive inflammatory responses. In CAR-T therapy, the modified T cells release large amounts of cytokines such as IL-6 and IFN-γ, triggering systemic inflammatory responses (cytokine release syndrome, CRS) [49,51]. This reaction increases vascular permeability, leading to hypotension and myocardial suppression, ultimately causing myocardial cell damage [50,52]. Additionally, CAR-T cells may directly attack myocardial cells, especially when the myocardial cells express proteins similar to the CAR-T target antigen, potentially causing myocarditis and leading to heart failure [49]. Excessive immune responses and cell damage can lead to cardiac toxicity in patients receiving CAR-T therapy.

### 2.4. Hormone Therapy Drugs

Hormone therapeutic drugs are commonly used to treat certain cancers by interfering with hormone synthesis or inhibiting tumor growth. Common hormone therapy drugs include estrogen receptor antagonists (such as tamoxifen) and aromatase inhibitors (such as anastrozole), which are primarily used to treat hormone-sensitive tumors such as breast and prostate cancers. These drugs target hormone receptors on the surface or inside tumor cells, thereby blocking hormone binding or inhibiting hormone production to reduce tumor proliferation.

#### 2.4.1. Anti-Estrogen and Anti-Androgen Drugs

Anti-estrogen and anti-androgen drugs function in cancer treatment by interfering with sex hormone signaling pathways to inhibit tumor growth. Anti-estrogen drugs such as tamoxifen and aromatase inhibitors reduce tumor cell growth by blocking estrogen from binding to its receptors or by inhibiting estrogen synthesis [111]. These drugs are particularly effective in treating estrogen receptor-positive (ER^+^) breast cancer [112]. Anti-androgen drugs such as flutamide and bicalutamide block the binding of androgens to their receptors, thereby inhibiting prostate cancer cell proliferation [113]. While these drugs are effective against hormone-dependent tumors, their use may be associated with cardiovascular risks. For instance, although the direct toxicity of tamoxifen to the heart is not fully understood, it is known to increase the risk of thrombosis, which may lead to cardiovascular events [53,54]. These potential adverse cardiovascular effects require close monitoring.

#### 2.4.2. Aromatase Inhibitors: Letrozole

Aromatase inhibitors (AIs) primarily reduce estrogen synthesis by inhibiting aromatase activity, particularly in postmenopausal women [114]. Aromatase is a key enzyme that converts androgens into estrogen and promotes tumor cell proliferation in ER^+^ breast cancer [115]. AI drugs reduce estrogen levels, decrease tumor dependence on hormones, and inhibit cancer cell growth and metastasis. Common aromatase inhibitors include letrozole, exemestane, and anastrozole. These drugs are especially effective in postmenopausal patients with breast cancer and reduce the risk of cancer recurrence with adjuvant therapy after surgery [115,116]. Despite their effectiveness, aromatase inhibitors may carry cardiovascular risks. Some studies indicate that they can lead to hyperlipidemia, which may increase the likelihood of developing cardiovascular disease [55,117]. Although the exact mechanism of cardiac toxicity is not yet fully understood, these drugs may have adverse effects on heart health.

### 2.5. Differentiation Inducers

Differentiation inducers play a crucial role in cancer therapy by promoting the differentiation of cancer cells into mature and functionally normal cells, thereby reducing their proliferative capacity and malignancy. Cancer cells are often in an undifferentiated or poorly differentiated state, possess high proliferative potential and abnormal gene expression, and lack the functionality of normal cells. Differentiation inducers stimulate cancer cells to differentiate into more mature cells with enhanced functionality and weaker proliferative abilities, ultimately inhibiting tumor growth [118]. These drugs typically activate specific cellular signaling pathways, prompting cancer cells to express genes related to differentiation, thereby driving the differentiation process.

For instance, vitamin A derivatives such as all-trans retinoic acid (ATRA) are common differentiation inducers. ATRA binds to retinoic acid receptors, activates differentiation genes, and promotes the differentiation of leukemia cells into mature blood cells [119]. This process not only reduces the proliferation of cancer cells but also increases their reliance on normal cellular behavior, lowering their invasiveness and metastatic potential [120]. Moreover, differentiation inducers can stimulate the expression of certain key enzymes and receptors in cancer cells, alter their function and biological behavior, and may even increase their sensitivity to other treatments such as chemotherapy and radiation therapy. These mechanisms make differentiation inducers highly effective in treating cancers, including acute promyelocytic leukemia (APL) [119].

Regarding the effects of ATRA on cardiac cells, studies have shown that ATRA may alleviate myocardial ischemia–reperfusion injury by inducing autophagy in myocardial cells [121]. However, these studies primarily focused on myocardial ischemia–reperfusion and did not establish the direct harmful effects of ATRA on cardiac cells. The use of high doses of ATRA may also influence cardiac development. Research suggests that ATRA can affect the structural development of myocardial tissues, particularly at specific doses [122]. Nevertheless, these results are mainly concerned with the developmental stages of the heart, and the direct damage to mature cardiac cells remains inconclusive. Further clinical and experimental studies are required to explore the potential effects of ATRA on cardiac cells.

## 3. Types and Potential Mechanisms of Mitochondria-Targeted Antioxidants

**Figure 2:**
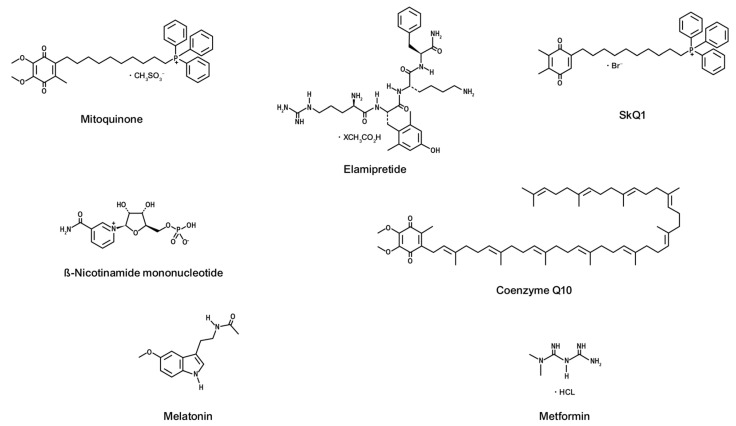
Chemical structures of mitochondria-targeted antioxidants.

Mitochondria-targeted antioxidants (MTAs) are a class of innovative drugs specifically designed to address oxidative stress within mitochondria and have potential therapeutic value in various cardiovascular diseases. Below are descriptions of some MTAs that are currently in use or under investigation (Figure 2).

### 3.1. MitoQ (Mitoquinone)

MitoQ selectively enters the mitochondria and scavenges excessive ROS, thereby protecting cells from oxidative stress-induced damage. MitoQ utilizes its triphenylphosphonium cation (TPP^+^) structure to cross the mitochondrial inner membrane and accumulate in the matrix, driven by high mitochondrial membrane potential [123,124]. The quinone portion of MitoQ acts as a potent antioxidant, reducing ROS to harmless water and alleviating oxidative stress [125,126]. Additionally, MitoQ neutralizes specific free radicals, reduces lipid peroxidation and DNA damage in the mitochondrial inner membrane, and helps stabilize mitochondrial membrane structure, maintain electron transport chain (ETC) function, improve energy production efficiency, and reduce ATP synthesis dysfunction [127,128]. These actions enable MitoQ to protect against various oxidative stress-related diseases, such as neurodegenerative disorders, cardiovascular diseases, and metabolic diseases [124]. Overall, MitoQ effectively reduces ROS generation and preserves mitochondrial structure and function through mitochondria-targeted antioxidant properties.

### 3.2. Elamipretide (SS-31, Bendavia)

Elamipretide (also known as SS-31 or Bendavia) is a mitochondria-targeted peptide antioxidant that primarily improves mitochondrial function and reduces oxidative stress damage [129]. Composed of four amino acids with both hydrophilic and hydrophobic properties, it can penetrate the cell membrane and localize to the inner mitochondrial membrane. Mitochondria are central to cellular energy metabolism. Excessive ROS generation leads to membrane lipid, protein, and DNA damage, thereby triggering apoptosis. Elamipretide binds to cardiolipin in the inner mitochondrial membrane, thereby stabilizing its structure [130]. Cardiolipins are prone to oxidation under oxidative stress, resulting in membrane disruption, reduced ETC efficiency, and diminished ATP production. Elamipretide prevents cardiolipin oxidation, protects ETC function, scavenges ROS, and reduces oxidative stress-induced damage to mitochondria and cells [130]. Additionally, elamipretide reduces abnormal mitochondrial membrane potential, prevents excessive calcium ion entry into mitochondria, and inhibits apoptotic pathways [130]. These effects enhance the efficiency of mitochondrial energy metabolism, stabilize ATP generation, and reduce oxidative damage. Elamipretide has demonstrated significant protective effects in various disease models, particularly in neurodegenerative, cardiovascular, and muscle-degenerative disorders associated with mitochondrial dysfunction [131].

### 3.3. SkQ1 (Plastoquinonyl Decyl Triphenylphosphonium)

SkQ1 primarily protects mitochondria by reducing ROS generation and neutralizing oxidative damage [132]. The molecular structure of SkQ1 includes lipophilic TPP^+^, a redox core plastoquinone, and alkyl chains, enabling it to effectively penetrate the cell membrane and accumulate in the negatively charged environment of the mitochondrial inner membrane [133]. The inner mitochondrial membrane is the primary site of the ETC, where electron leakage generates ROS, such as superoxide anions. Excess ROS can damage membrane lipids, proteins, and DNA, thereby impairing the mitochondrial function and inducing cell death. SkQ1 undergoes redox cycling through its plastoquinone portion, directly neutralizing superoxide anions and other ROS while protecting cardiolipin from oxidative damage, stabilizing the mitochondrial inner membrane structure, and maintaining ETC function [133]. This action helps reduce ROS generation, preserves mitochondrial health, and inhibits oxidative stress-induced mitochondrial membrane potential abnormalities and excessive calcium ion entry into the mitochondria, thereby preventing apoptosis signaling. SkQ1 has shown potential in animal models and cell studies, demonstrating its ability to slow aging, neurodegenerative diseases, and cardiovascular diseases, proving its protective effects on mitochondrial function and cell survival [133,134].

### 3.4. Nicotinamide Mononucleotide (NMN) and NAD^+^ Enhancers

Nicotinamide mononucleotide (NMN) and NAD^+^ enhancers promote cellular metabolism, energy production, and mitochondrial function by increasing the intracellular NAD^+^ levels. NAD^+^ is a crucial coenzyme involved in energy metabolism, DNA repair, and cellular signaling. NAD^+^ levels significantly decrease with aging and in certain pathological conditions, affecting mitochondrial function and causing cellular damage [135]. NMN, a direct precursor of NAD^+^, is rapidly absorbed by cells and converted into NAD^+^, effectively replenishing NAD^+^ levels within cells to support the tricarboxylic acid cycle and ETC, promoting ATP production [136].

NAD^+^ is also a substrate for various enzymes such as sirtuins and PARPs. Sirtuins (SIRT1 and SIRT3) are NAD^+^-dependent deacetylases that play essential roles in mitochondrial biogenesis, antioxidant defense, and metabolic health [137]. NMN increases NAD^+^ levels, activates sirtuins to improve mitochondrial function, and reduces oxidative stress-induced damage [138]. NAD^+^ promotes PARP activity, contributes to DNA repair, maintains genomic stability, and delays cell aging [137].

NMN and NAD^+^ enhancers also support cellular antioxidant capacity, reduce ROS generation, and protect the inner mitochondrial membrane from damage. In summary, NMN and NAD^+^ enhancers promote energy metabolism, enhance antioxidant defense, improve mitochondrial function, and have the potential to treat healthy aging, neurodegenerative diseases, and metabolic disorders [136,139,140].

### 3.5. Coenzyme Q10 (CoQ10)

CCoenzyme Q10 (CoQ10), also known as ubiquinone, is a naturally occurring lipophilic antioxidant found in human cells that is crucial for maintaining cellular energy metabolism and protecting cells from oxidative damage [141]. CoQ10 primarily functions in the mitochondrial ETC, where it acts as an electron carrier, transferring electrons from complexes I and II to complex III, driving proton pumping, establishing a membrane electrochemical gradient, and facilitating ATP synthesis to provide energy to cells [142].

Additionally, CoQ10 exhibits potent antioxidant properties, neutralizes free radicals, reduces ROS generation, and inhibits oxidative stress-induced damage [143]. It captures free radicals, prevents lipid peroxidation of cell membranes, and regenerates other antioxidants such as vitamins E and C, thereby enhancing cellular antioxidant defenses [143]. CoQ10 also stabilizes the mitochondrial inner membrane structure, protecting the mitochondria from oxidative damage, thereby maintaining normal cellular function [144].

CoQ10 modulates the cell death process by inhibiting the excessive opening of mitochondrial permeability transition pores (mPTP) to prevent apoptosis, thus preventing cells from undergoing programmed death due to excessive stress [145,146]. It also participates in anti-inflammatory responses, reduces the release of inflammatory cytokines, and mitigates cell damage caused by chronic inflammation [147]. CoQ10 has a potential therapeutic value in energy metabolism disorders, neurodegenerative diseases, cardiovascular diseases, and age-related pathologies [148].

### 3.6. Melatonin

Melatonin is an endogenous hormone secreted by the pineal gland that plays an essential role in regulating biological rhythms, antioxidant defense, and cellular protection [149]. Melatonin is a mitochondria-targeted antioxidant that effectively reduces oxidative stress, protects mitochondrial function, and maintains cellular homeostasis [150]. It directly scavenges free radicals (such as superoxide anions and hydroxyl radicals) and reactive nitrogen species (such as peroxynitrite), thereby reducing oxidative stress-induced cellular damage [150].

Moreover, melatonin regulates the antioxidant enzyme system, promoting the expression and activity of antioxidant enzymes such as superoxide dismutase (SOD) and catalase (CAT), and inhibiting pro-oxidative enzymes such as nitric oxide synthase (NOS), thereby enhancing cellular antioxidant defense [151]. These actions effectively reduce free radical damage to cellular structures, particularly the mitochondrial membranes and DNA.

In terms of mitochondrial function, melatonin stabilizes the mitochondrial membrane potential, inhibits excessive opening of the mPTP, and prevents apoptosis [152]. It also reduces mitochondrial calcium overload, maintains energy metabolism stability, and protects respiratory chain complexes, thereby promoting normal ATP production [153].

In summary, melatonin provides multilayered protection to cells through antioxidant activity, protection of mitochondrial function, and regulation of cell death, offering potential therapeutic value for neurodegenerative diseases, cardiovascular diseases, metabolic disorders, and aging-related diseases [154].

### 3.7. Metformin

Metformin is commonly used to treat type 2 diabetes mellitus, and recent studies have investigated its potential as a mitochondria-targeted antioxidant [155]. Its primary mechanisms of action include inhibition of mitochondrial ETC complex I, reduction in ROS generation, activation of AMP-activated protein kinase (AMPK), and improvement in cellular energy metabolism, providing multiple protective effects [156]. Metformin reduces excessive electron leakage and ROS generation by inhibiting complex I and alleviating oxidative stress-induced cellular damage, particularly in pathological conditions such as metabolic disorders and aging [157].

Furthermore, metformin lowers the mitochondrial membrane potential, prevents excessive opening of mitochondrial permeability transition pores, protects mitochondrial function, and prevents apoptosis triggered by stress [158]. It also reduces calcium ion overload in the mitochondria, further stabilizing mitochondrial structure. In terms of cellular metabolism, metformin activates AMPK, promoting glucose uptake, fatty acid oxidation, and mitochondrial biogenesis, thereby improving overall energy metabolism [159]. The activation of AMPK also suppresses the mTOR pathway associated with oxidative stress, reducing cellular aging and pathological conditions [160].

Metformin promotes mitochondrial autophagy, clears damaged mitochondria, and maintains cellular health [161]. In conclusion, through multiple mechanisms, metformin not only plays a key role in diabetes treatment but also holds potential as a therapeutic drug for delaying aging and treating oxidative stress-related conditions, such as neurodegenerative diseases [162].

## 4. Cardioprotective Potential of MTAs Against Chemotherapy-Induced Cardiotoxicity

MTAs have shown significant potential for protecting the heart from chemotherapy-induced cardiotoxicity, primarily by reducing oxidative stress, preserving mitochondrial function, and stabilizing cellular energy metabolism. Chemotherapeutic agents such as anthracyclines often cause mitochondrial damage and myocardial cell death by generating excessive ROS. MTAs such as MitoQ, elamipretide, and CoQ10 act directly on the mitochondria to scavenge excess ROS, inhibit the opening of the mPTP, and protect the integrity of the mitochondrial membrane. These antioxidants also regulate mitochondrial dynamics, promote mitochondrial autophagy to eliminate damaged mitochondria, and sustain the energy supply to myocardial cells. Furthermore, they suppress the release of inflammatory mediators and activation of apoptotic pathways, thereby mitigating the cardiotoxic effects induced by chemotherapy. Therefore, MTAs hold promise as adjunctive therapies for improving chemotherapy tolerance and reducing adverse cardiovascular effects. The following Table 2 summarizes some MTAs that have shown potential for alleviating chemotherapy-induced cardiotoxicity.

### 4.1. Protective Effects of MitoQ

#### 4.1.1. Effects on ROS Generation and Oxidative Stress

One of the main mechanisms by which MitoQ alleviates chemotherapy-induced cardiotoxicity is by reducing ROS generation, thereby mitigating oxidative stress [172]. Chemotherapeutic drugs, such as doxorubicin, trigger a significant increase in intracellular ROS, which damages the mitochondria, leading to myocardial cell dysfunction and heart failure. Studies have shown that MitoQ can effectively enter cardiac cells and act directly on the mitochondria to reduce ROS production [134]. In animal experiments, mice treated with MitoQ showed a significant reduction in oxidative stress in the heart after doxorubicin treatment, with lower ROS levels in myocardial cells than in untreated controls, thereby reducing the associated myocardial damage [173]. This action of MitoQ helps reduce oxidative damage to myocardial cells, protecting the heart from chemotherapy-induced toxicity.

#### 4.1.2. Effects on Mitochondrial Function and Structural Protection

MitoQ prevents chemotherapy-induced cardiotoxicity by improving mitochondrial function, particularly by protecting the mitochondrial membranes. Animal studies have shown that MitoQ reduces mitochondrial membrane damage caused by chemotherapy drugs, preserves the mitochondrial structure within myocardial cells, and enhances cellular energy production [163]. Additionally, MitoQ promotes mitochondrial autophagy, helping to eliminate damaged mitochondria, which reduces sources of oxidative damage, maintains the normal function of myocardial cells, and improves overall cardiac function [183].

#### 4.1.3. Effects on Myocardial Inflammatory Response and Cell Apoptosis

Several animal studies have demonstrated that MitoQ effectively reduces chemotherapy-induced inflammation. In these experiments, MitoQ significantly suppressed the expression of pro-inflammatory cytokines (such as TNF-α and IL-6) in myocardial cells, which are known to play crucial roles in chemotherapy-induced cardiac injury [184]. MitoQ also reduces apoptosis in myocardial cells [185]. MitoQ significantly reduced myocardial cell apoptosis in animals treated with doxorubicin, thereby effectively reducing chemotherapy-induced myocardial damage [163].

#### 4.1.4. Effects on Calcium Ion Homeostasis and Cellular Function

Chemotherapy-induced oxidative stress and mitochondrial damage can lead to abnormalities in calcium ion storage and release in the myocardial cells, thereby impairing heart function. MitoQ regulates calcium ion homeostasis [186]. Animal studies have shown that MitoQ can balance intracellular calcium ions in myocardial cells, reducing the calcium overload caused by oxidative damage, which plays a critical role in reducing cardiac dysfunction [180,181,187].

Overall, as a mitochondria-targeted antioxidant, MitoQ alleviates chemotherapy-induced cardiotoxicity through multiple mechanisms, including the reduction in ROS generation, protection of mitochondrial function, reduction in inflammation, and regulation of calcium ion stability. In both cellular and animal studies, MitoQ has demonstrated potential as a cardioprotective agent, providing strong support for its clinical application.

### 4.2. Protective Effects of Elamipretide

#### 4.2.1. Effects on Mitochondrial Function Protection and Oxidative Stress Suppression

Elamipretide improves the structure and function of damaged mitochondria and reduces ROS generation. In animal experiments, heart cells treated with elamipretide showed significant restoration of mitochondrial function and a significant reduction in oxidative stress compared to the untreated groups [165]. This indicates that elamipretide effectively reduces the oxidative damage induced by chemotherapy drugs, thus protecting the heart from toxic effects.

#### 4.2.2. Effects on Mitochondrial Membrane Stability

Another mechanism of Elamipretide is the stabilization of the mitochondrial membrane, reducing membrane damage. Research has shown that elamipretide can promote mitochondrial membrane repair, preventing changes in membrane permeability caused by chemotherapy drugs, thereby reducing energy deficiency [164,165]. By stabilizing the mitochondrial membrane potential, it helps maintain the normal function of myocardial cells, thus reducing the occurrence of cardiotoxicity.

#### 4.2.3. Effects on Myocardial Cell Apoptosis and Inflammatory Response

Elamipretide reduces apoptotic signaling in myocardial cells by inhibiting chemotherapy-induced cell death. Studies have found that compared with the control group, the number of apoptotic cells in the heart tissue of animals treated with elamipretide was significantly reduced [165]. In addition, elamipretide reduces the inflammatory response induced by chemotherapy drugs, which often exacerbate cardiac injury. In experiments, the levels of inflammatory factors were significantly lower in the tissues of animals treated with elamipretide, indicating that it can inhibit the inflammatory response induced by chemotherapy, further alleviating cardiac damage [178].

#### 4.2.4. Effects on Cardiac Contractile Function and Structural Protection

In animal models, elamipretide significantly improved cardiac contractile function. Chemotherapeutic drugs (such as doxorubicin) often cause cardiac contractility dysfunction, leading to heart failure. The experimental results showed that elamipretide could protect the structure and function of the heart, thereby improving its pumping efficiency. Animals treated with elamipretide showed a better left ventricular ejection fraction (LVEF) and left ventricular end-diastolic volume (LVEDV) on echocardiography, indicating that heart function was not severely affected by chemotherapy. Furthermore, histological examination of heart tissues revealed that elamipretide-treated animals had intact myocardial fiber structures with no significant edema or fibrosis, indicating its protective effect on cardiac structure [129].

#### 4.2.5. Effects on Intracellular Calcium Ion Balance

Elamipretide improves the stability of intracellular calcium ions in the myocardial cells and prevents calcium overload. Studies have shown that in animals treated with chemotherapeutic drugs, elamipretide regulates the calcium ion concentration within myocardial cells, reducing cell damage caused by calcium overload [182]. This mechanism protects myocardial cells and reduces chemotherapy-induced cardiotoxicity.

In summary, elamipretide, a mitochondria-targeting antioxidant, demonstrated significant cardioprotective potential against chemotherapy-induced cardiotoxicity. Through various mechanisms, such as reducing oxidative stress, protecting mitochondrial function, reducing apoptosis, inhibiting inflammation, and regulating calcium ion balance, it significantly reduces cardiac damage caused by chemotherapy drugs. These results provide strong scientific evidence for its potential use as an adjunct therapy for chemotherapy-induced cardiotoxicity.

### 4.3. Protective Effects of CoQ10

#### 4.3.1. Protection of Mitochondrial Function and Energy Metabolism

CoQ10 counteracted toxic reactions by improving mitochondrial function. In animal experiments, the heart tissues from the CoQ10-treated group showed significantly higher ATP levels and preserved mitochondrial structural integrity [166]. This suggests that CoQ10 supports the energy demands of myocardial cells, thereby ameliorating the decline in heart function induced by chemotherapeutic drugs.

#### 4.3.2. Inhibition of Oxidative Stress

As a potent antioxidant, CoQ10 effectively reduced the production of harmful oxidative molecules. Both cell and animal studies have shown that CoQ10 treatment significantly decreases ROS production induced by chemotherapy drugs, and markers of oxidative damage (such as MDA and 4-HNE) in myocardial cells are notably reduced [171,175]. These results indicated that CoQ10 protects myocardial cells from chemotherapy-induced damage by inhibiting oxidative stress.

#### 4.3.3. Inhibition of Apoptotic Pathways

CoQ10 reduces apoptosis triggered by oxidative stress. Research indicates that in animal heart tissues treated with CoQ10, apoptotic markers (such as Caspase-3 and Bax) are significantly decreased, whereas anti-apoptotic factors (such as Bcl-2) are upregulated [171,174]. These findings suggest that CoQ10 can inhibit myocardial cell death by regulating apoptosis-related proteins, thereby reducing heart damage caused by chemotherapy drugs.

#### 4.3.4. Inhibition of Inflammatory Responses

CoQ10’s protective effect on the heart is also reflected in its ability to suppress the inflammation caused by chemotherapeutic drugs. The experimental results show that animals treated with CoQ10 exhibited significantly lower levels of inflammatory markers (such as TNF-α, IL-6, IL-1β) in their hearts following chemotherapy [179]. These findings suggest that CoQ10 alleviates cardiac toxicity by reducing inflammation.

#### 4.3.5. Protection of Cardiac Structure and Function

In animal experiments, CoQ10-treated animals showed no signs of heart edema, fibrosis, or other structural damage caused by chemotherapy drugs, and heart function indicators (such as LVEF and end-diastolic volume) were not significantly affected [188]. This suggests that CoQ10 protects myocardial cells and the overall structure and function of the heart during chemotherapy-induced cardiotoxicity.

#### 4.3.6. Clinical Trial

Clinical trials have also confirmed CoQ10’s potential in protecting the heart. In a study involving patients with breast cancer undergoing chemotherapy, those who received CoQ10 supplementation showed no significant decline in heart function, and their echocardiographic parameters remained normal. Compared with the control group, patients receiving CoQ10 had a significantly lower incidence of heart toxicity and milder heart-related symptoms (such as fatigue and shortness of breath) [167,168,189]. This indicates that CoQ10 exerts protective effects against chemotherapy-induced cardiac toxicity.

In summary, CoQ10 has demonstrated significant cardioprotective potential in various cell, animal, and human trials. It can reduce chemotherapy-induced cardiac toxicity through multiple mechanisms such as reducing oxidative stress, inhibiting inflammation, protecting mitochondrial function, reducing apoptosis, and maintaining heart structure and function. These findings provide evidence for CoQ10’s potential as an adjunct treatment for chemotherapy-induced cardiotoxicity.

### 4.4. Protective Effects of Melatonin

#### 4.4.1. Inhibition of Oxidative Stress

As a powerful antioxidant, melatonin has been shown in various animal and cellular studies to effectively reduce ROS production induced by chemotherapy drugs. In animal studies, melatonin significantly decreases oxidative stress markers (such as MDA and 8-OHdG) in myocardial tissue and increases the activity of antioxidant enzymes, such as SOD and CAT [169,176]. These studies suggest that melatonin reduces oxidative damage to cardiac cells, thereby protecting the myocardium from chemotherapy-induced toxicity.

#### 4.4.2. Protection of Mitochondrial Function

In animal studies, hearts from melatonin-treated animals showed preserved mitochondrial structural integrity and a significant recovery of ATP levels. Further research indicated that melatonin promotes mitochondrial membrane stability and reduces chemotherapy-induced mitochondrial collapse [170]. This helps prevent energy depletion and functional impairment in myocardial cells.

#### 4.4.3. Inhibition of Apoptotic Pathways

Melatonin has been shown to effectively reduce chemotherapy-induced apoptosis of myocardial cells in several cell and animal models. Melatonin treatment significantly reduced the levels of pro-apoptotic proteins such as Caspase-3 and Bax, whereas anti-apoptotic proteins such as Bcl-2 were upregulated [169,170,176]. These results suggest that melatonin reduces cell death by regulating apoptosis-related proteins, which helps reduce heart damage and maintain cardiac function.

#### 4.4.4. Inhibition of Inflammatory Responses

Melatonin has been shown to suppress chemotherapy-induced cardiac inflammation in animal studies. Specifically, melatonin significantly reduced levels of pro-inflammatory cytokines (such as TNF-α, IL-6, IL-1β) and inflammatory markers (such as C-reactive protein, CRP) in heart tissue [169]. These findings suggested that melatonin protects the heart by reducing inflammation during chemotherapy-induced cardiac toxicity.

#### 4.4.5. Protection of Cardiac Structure and Function

In animal models, melatonin treatment resulted in lower heart edema and fibrosis after chemotherapy (e.g., doxorubicin treatment), and heart function indicators (such as ejection fraction and LVEDV) showed no significant decline [169,176]. This suggests that melatonin protects the heart structure and function, effectively preventing chemotherapy-induced cardiotoxicity.

In conclusion, melatonin has been shown to reduce chemotherapy-induced cardiac toxicity in cellular, animal, and clinical studies. It protects the heart through multiple mechanisms, including reduction in oxidative stress, protection of mitochondrial function, inhibition of apoptosis, reduction in inflammation, and preservation of heart structure and function. These results strongly support melatonin as a potential adjunct therapy for chemotherapy-induced cardiotoxicity.

### 4.5. Protective Effects of SkQ1

#### 4.5.1. Inhibition of Oxidative Stress and ROS Generation

SkQ1, through its unique plastoquinone structure, directly scavenges excessive ROS in mitochondria and reduces oxidative stress induced by chemotherapy drugs (e.g., anthracyclines). Research has shown that in cell and animal models of chemotherapy-induced toxicity, SkQ1 significantly reduces superoxide anion and hydrogen peroxide levels in myocardial cells, preventing lipid peroxidation damage to cell membranes [134]. This not only reduces myocardial cell necrosis and apoptosis but also improves mitochondrial function stability.

#### 4.5.2. Reduction in Mitochondria-Related Apoptosis

SkQ1 has been shown to effectively reduce chemotherapy-induced damage in H9c2 myocardial cells [177]. SkQ1 significantly improved cell viability in H9c2 cells exposed to Dox. SkQ1 reduced oxidative stress markers and preserved mitochondrial membrane potential, highlighting their efficacy in mitigating Dox-induced cellular and mitochondrial damage.

#### 4.5.3. Improvement in Inflammatory Responses

Both in vitro and in vivo studies show that SkQ1 reduces myocardial inflammation markers, significantly decreasing the degree of heart inflammation following hemorrhagic shock [190].

In summary, SkQ1 protects the heart primarily by scavenging mitochondrial ROS, stabilizing mitochondrial function, inhibiting apoptosis, and reducing inflammation. Significant cardioprotection has been demonstrated in cell and animal studies, providing a promising therapeutic option to prevent chemotherapy-related cardiac toxicity.

## 5. Future Perspectives

In recent years, research on chemotherapy-induced myocardial injury has increasingly focused on the application of MTAs. Chemotherapy drugs, such as anthracyclines like doxorubicin, can cause myocardial cell damage through mechanisms including oxidative stress, mitochondrial dysfunction, and ferroptosis, ultimately leading to cardiotoxicity. While conventional antioxidants can mitigate free radical damage to some extent, their lack of specificity makes it difficult for them to effectively reach mitochondria and exert their protective effects.

MTAs, such as MitoQ and SkQ1, contain a positively charged TPP group, allowing them to actively accumulate in mitochondria via the MMP. These antioxidants can directly neutralize ROS, reduce oxidative stress, and protect cardiomyocytes. Additionally, they help maintain MMP stability, regulate apoptotic pathways, and reduce cardiomyocyte apoptosis, thereby alleviating chemotherapy-induced myocardial injury. Furthermore, advances in nanotechnology have further enhanced the efficacy of MTAs. By incorporating these antioxidants into nanoparticle carriers with cardiac-targeting and anchoring capabilities, their stability can be improved, and their selective distribution in cardiac tissues can be increased, thereby enhancing cardioprotective effects [191].

Despite their promising potential in preventing and treating chemotherapy-induced myocardial injury, MTAs require further research to validate their clinical application. First, most current studies are still limited to cell experiments or animal models, with limited clinical trial data. Therefore, more randomized controlled trials are needed to evaluate their safety, efficacy, and optimal treatment protocols. Second, further improvements in drug targeting and minimizing potential side effects remain key research priorities.

Moreover, combination therapy strategies involving MTAs represent another promising avenue for advancing treatment. For instance, these antioxidants could be used in combination with existing cardioprotective agents, such as β-blockers, ACE inhibitors, or iron chelators, to achieve enhanced protective effects [192,193,194,195]. Additionally, advances in artificial intelligence and precision medicine may aid in developing personalized treatment plans, improving therapeutic efficacy while reducing the risk of adverse effects.

## 6. Conclusions

MTAs show significant potential in reducing chemotherapy-induced cardiac toxicity, offering new possibilities for clinical treatment. Chemotherapy drugs, particularly anthracyclines, platinum-based drugs, and fluorouracil, are crucial for cancer treatment but also cause a range of cardiac side effects, including myocardial damage and heart failure. These side effects often result from chemotherapeutic drugs that damage the mitochondria, leading to increased oxidative stress, myocardial cell damage, and impaired heart function. Thus, antioxidants targeting the mitochondria have shown great potential as cardioprotective agents, reducing chemotherapy-induced heart toxicity.

Mitochondria are the core of cellular energy metabolism and the primary site of free radical generation. Oxidative damage caused by chemotherapy drugs is often associated with impaired mitochondrial function, leading to energy depletion, excessive oxidative stress, and apoptosis. Mitochondria-targeted antioxidants can effectively scavenge excess free radicals, reduce oxidative stress-induced damage to the heart, and protect the mitochondrial structure and function. Antioxidants such as elamipretide, SkQ1, CoQ10, and melatonin have demonstrated cardiac protection in various animal models.

**Figure 3:**
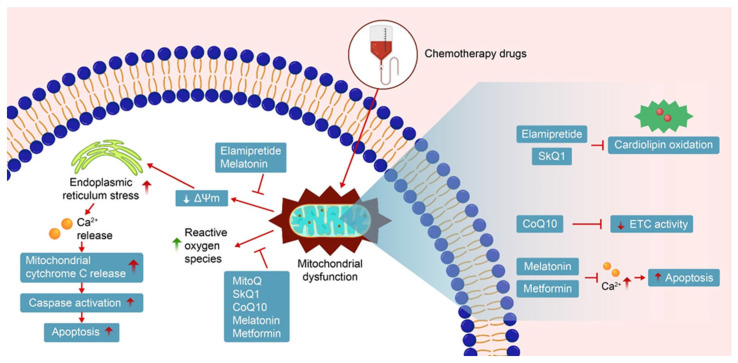
Mitochondrial-targeted antioxidants based protection against chemotherapy-induced cardiotoxicity.

The mechanisms of action of these antioxidants typically include the direct neutralization of free radicals, stabilization of mitochondrial membrane structures, inhibition of the opening of mitochondrial permeability transition pores, and protection of myocardial cells from damage. In addition, many mitochondrial-targeted antioxidants activate antioxidant enzyme systems, enhancing the cell’s antioxidant capacity, and promote mitochondrial autophagy to remove damaged mitochondria, preventing further cell damage (Figure 3).

Although these drugs have shown strong protective effects in laboratory and animal studies, further clinical validation is needed. Future studies should focus on improving the stability and bioavailability of these antioxidants, determining their optimal dosages and administration routes, and ensuring maximum efficacy. In clinical settings, mitochondria-targeted antioxidants are expected to become a new option for reducing chemotherapy-induced cardiac toxicity and improving the quality of life of patients.

Overall, mitochondria-targeted antioxidants exhibit promising therapeutic potential for combating chemotherapy-induced cardiac toxicity, providing an effective pathway to reduce heart damage. As research progresses, these therapies are expected to become key components of clinical practice, significantly enhancing the safety and long-term survival of patients with cancer. Currently, clinical trials investigating the combined use of anticancer drugs and antioxidants remain relatively limited, and their outcomes remain inconclusive. Some studies have explored the potential benefits of such combination therapies, suggesting that antioxidants may help reduce chemotherapy-induced oxidative stress and tissue damage, thereby minimizing side effects and improving patients’ quality of life. However, there are concerns that antioxidants might protect cancer cells, potentially reducing the efficacy of treatment. As a result, the advantages and necessity of this approach—combining anticancer drugs with antioxidants—have yet to be fully established, and further clinical trials are needed to confirm their efficacy and safety.

In conclusion, MTAs offer an innovative therapeutic strategy for chemotherapy-induced myocardial injury. With ongoing technological advancements and further clinical research, these drugs have the potential to serve as an important adjunct therapy in cancer treatment, helping to mitigate cardiotoxicity and improve both treatment outcomes and the quality of life for cancer patients.

## Figures and Tables

**Table 1:** Types of chemotherapeutic agents and potential mechanisms of myocardial injury.

Drug Category	Drug Subcategory	Related Drugs	Mechanism of Action	Mechanisms of Cardiac Injury	References
Cytotoxic Drugs	Alkylasting Agents	Cyclophosphamide, Cisplatin	Bind to DNA, form cross-links, and interfere with DNA replication and transcription.	Toxicity of metabolites, oxidative stress, DNA damage, and inflammatory responses.	[8,18,19,20,21]
	Antimetabolites	Methotrexate, 5-Fluorouracil	Mimic nucleotides, disrupting DNA or RNA synthesis.	Coronary artery spasm, toxicity of metabolites, oxidative stress, microvascular damage.	[22,23,24,25,26,27,28]
	Natural Products	Taxanes, Vinca Alkaloids	Affect microtubule structures, inhibit cell division.	Arrhythmias, allergic reactions, microtubule dysfunction, neurotoxicity, bone marrow suppression.	[20,29,30,31]
		Antibiotics (Anthracyclines)	Interfere with DNA function and generate free radicals.	Oxidative stress and free radical generation, iron chelation and toxic metabolites, DNA damage and cell apoptosis	[10,32,33,34]
Targeted Therapy	Tyrosine Kinase Inhibitors	Imatinib, Erlotinib	Inhibit signaling pathways required for cancer cell growth.	Endoplasmic reticulum (ER) stress and inflammatory responses, mitochondrial dysfunction, endothelial cell damage, QT interval prolongation.	[35,36,37,38]
	Monoclonal Antibodies	Trastuzumab, Bevacizumab	Target specific receptors or antigens on the cell surface.	HER2 receptor blockage, inhibition of vascular endothelial growth factor (VEGF), heart strain induced by hypertension.	[39,40,41,42,43]
Immunotherapy	Immune Checkpoint Inhibitors	Pembrolizumab, Ipilimumab	Block immune checkpoint pathways such as PD-1 or CTLA-4.	Immune-mediated myocarditis, inhibition of the PD-1/PD-L1 pathway in cardiomyocytes, release of inflammatory cytokines.	[44,45,46,47,48]
	Cell Therapy		Chimeric Antigen Receptor T-Cell Therapy (CAR-T)	Cytokine release syndrome (CRS), direct myocarditis, vascular dysfunction.	[49,50,51,52]
Hormone Therapy	Anti-estrogen/androgen Drugs	Tamoxifen, Bicalutamide	Target hormone-sensitive tumors (e.g., breast cancer, prostate cancer) by inhibiting or reducing hormone production.	Thrombosis, lipid metabolism issues (e.g., hyperlipidemia).	[53,54,55]
	Aromatase Inhibitors	Letrozole			
Other	Differentiation Inducers	All-trans retinoic acid (ATRA)	Induces cancer cells to differentiate into normal cells.	Not determined	

**Table 2:** Cardioprotective potential of MTAs against chemotherapy-induced cardiotoxicity.

Target	Drug Type	Experimental Model	References
Mitochondrial Function and Energy Metabolism Protection	MitoQ, Elamipretide, Coenzyme Q10 (CoQ10), Melatonin, SkQ1	Animal testing/Human trails	[163,164,165,166,167,168,169,170,171]
Inhibition of Oxidative Stress	MitoQ, Elamipretide, CoQ10, Melatonin, SkQ1	Cell experiments/Animal testing	[134,165,169,171,172,173,174,175,176,177]
Myocardial Inflammation and Cell Apoptosis	MitoQ, Elamipretide, CoQ10, Melatonin, SkQ1	Animal testing	[163,169,171,174,176,177,178,179]
Calcium Ion Stability	MitoQ, Elamipretide	Cell experiments	[180,181,182]

## Abbreviations

The following abbreviations are used in this manuscript:

5-FU	Fluorouracil
ADCC	Antibody-dependent cellular cytotoxicity
ALL	Acute lymphoblastic leukemia
ATP	Adenosine triphosphate
APL	Acute promyelocytic leukemia
ATRA	All-trans retinoic acid
CARs	Chimeric antigen receptors
CAT	Catalase
CoQ10	Coenzyme Q10
CRP	C-reactive protein
CRS	Cytokine release syndrome
ETC	Electron transport chain
ER	Endoplasmic reticulum
ER^+^	Estrogen receptor-positive
ICIs	Immune checkpoint inhibitors
IL-6	Interleukin-6
LVEF	Left ventricular ejection fraction
MitoQ	Mitoquinone
mPTP	Mitochondrial permeability transition pores
MTAs	Mitochondria-targeted antioxidants
NK	Natural killer
NMN	Nicotinamide Mononucleotide
NOS	Nitric oxide synthase
ROS	Reactive oxygen species
SOD	Superoxide dismutase
TNF-α	Tumor necrosis factor-α
TKIs	Tyrosine kinase inhibitors
TPP^+^	Triphenylphosphonium cation
VEGF	Vascular endothelial growth factor
